# Below-elbow cast sufficient for treatment of minimally displaced metaphyseal both-bone fractures of the distal forearm in children: long-term results of a randomized controlled multicenter trial

**DOI:** 10.1080/17453674.2021.1889106

**Published:** 2021-02-22

**Authors:** Linde Musters, Leon W Diederix, Kasper C Roth, Pim P Edomskis, Gerald A Kraan, Jan H Allema, Max Reijman, Joost W Colaris

**Affiliations:** a Department of Orthopedics, Noordwest Ziekenhuisgroep Alkmaar , The Netherlands ;; b Department of Orthopedics, Elkerliek Hospital, Helmond;;; c Department of Orthopedics, Erasmus MC, University Medical Centre , Rotterdam ;; d Department of Surgery, Erasmus MC, University Medical Centre , Rotterdam ;; e Department of Orthopedics, Reinier de Graaf , Delft ;; f Department of Surgery, Haga Hospital , The Hague , The Netherlands

## Abstract

Background and purpose — We have previously shown that children with minimally displaced metaphyseal both-bone forearm fractures, who were treated with a below-elbow cast (BEC) instead of an above-elbow cast (AEC), experienced more comfort, less interference in daily activities, and similar functional outcomes at 7 months’ follow-up (FU). This study evaluates outcomes at 7 years’ follow-up.

Patients and methods — A secondary analysis was performed of the 7 years’ follow-up data from our RCT. Primary outcome was loss of forearm rotation compared with the contralateral forearm. Secondary outcomes were patient-reported outcome measures (PROMs) consisting of the ABILHAND-kids and the DASH questionnaire, grip strength, radiological assessment, and cosmetic appearance.

Results — The mean length of FU was 7.3 years (5.9–8.7). Of the initial 66 children who were included in the RCT, 51 children were evaluated at long-term FU. Loss of forearm rotation and secondary outcomes were similar in the 2 treatment groups.

Interpretation — We suggest that children with minimally displaced metaphyseal both-bone forearm fractures should be treated with a below-elbow cast.

Long-term follow-up of children with forearm fractures is scarce but essential, because the remodeling capacity by growth can behave as a friend or an enemy. Previous studies with short-term follow-up shown that metaphyseal both-bone fractures of the distal forearm could safely be treated with a below-elbow cast (BEC) (Bohm et al. 2006, Webb et al. 2006, Paneru et al. 2010, Hendrickx et al. 2011, Colaris et al. 2012, Van Den Bekerom et al. 2012). Our previous randomized multicenter controlled trial compared BEC with above-elbow cast (AEC) for the treatment of minimally displaced metaphyseal both-bone fractures of the distal forearm in children. This RCT concluded that children with minimally displaced metaphyseal both-bone fractures of the distal forearm should be treated with a below-elbow cast (Colaris et al. 2012). We now report the long-term 7-year follow-up of these 2 treatment groups regarding loss of forearm rotation, patient-reported outcomes measures (ABILHAND-kids questionnaire and DASH questionnaire (Hudak et al. 1996, Penta et al. 1998, Arnould et al. 2004), grip strength, radiological assessment, and cosmetic appearance (Bohm et al. 2006, Paneru et al. 2010, Hendrickx et al. 2011, Colaris et al. 2012, Van Den et al. 2012)

## Patients and methods

### Trial design and participants

All patients who had been previously included between 2006 and 2010 in the RCT were invited to our outpatient clinic to determine long-term clinical outcomes with a minimum follow-up of 5 years (Colaris et al. 2012). These patients had been children with a minimally displaced metaphyseal fracture of the radius and ulna, who had been randomized between treatment with AEC or BEC. Informed consent was again obtained from all participants and from all the parents of children aged < 12 years.

### Outcomes measures

Our primary outcome measure was loss of forearm rotation in comparison with the contralateral side. Secondary outcome measures were patient-reported outcome measures (PROMs): using the Dutch version of the DASH and ABILHAND-kids questionnaire, wrist and elbow range of motion, grip strength (using a JAMAR Dynamometer), VAS scores regarding cosmetic appearance (scars and angulation of the forearm), and radiological assessment of malunion (Hudak et al. 1996, Penta et al. 1998, Arnould et al. 2004).

An orthopedic surgeon (LD) measured forearm rotation, flexion, and extension of wrist and elbow using visual estimation and a goniometer with increments of 2°. The follow-up was organized in the patient’s original hospital of inclusion. Both arms were examined to determine functional loss. Grip strength was measured using a Jamar dynamometer (Performance Health International, Sutton-in-Ashfield, UK), conducting one measurement comparing both arms. Patients were asked to fill in 2 PROMs, the DASH and the ABILHAND-kids questionnaire, and a VAS for cosmetic appearance. Cosmetic appearance was assessed by the patient, or by the parents in children < 12 years, and by the investigator (LD).

The radiological assessment consisted of a anteroposterior and lateral radiograph of the wrist. One of the authors (PE), measured the angulation of the radius and ulna (Zimmermann et al. 2004, Jeroense et al. 2015).

### Statistics

To evaluate whether the included patients in the current study are representative of the total initial study population of 66 patients, we compared the baseline characteristics, functional outcome, and complications at short-term follow-up (7 months) between the included patients versus those lost to follow-up. Long-term results of primary and secondary outcome measures of the 2 treatment groups (AEC vs. BEC) were compared. Differences were analyzed using 1-way ANOVA to correct for multiple comparisons. Results are presented as mean SD or 95% confidence interval (CI). To assess the inter-rater reproducibility of radiographic assessment 2 authors (PE and LD) measured angulations of the radius and ulna of 25 cases (at cast removal and at final follow-up). Intra-class correlation coefficient was calculated. Statistical analyses were performed using IBM SPSS Statistics version 23.

### Ethics, registration, funding, and potential conflict of interest

Ethics approval was obtained for this post-trial FU study from regional medical ethics committee (NL41839.098.12). The original RCT was registered in ClinicalTrials.gov NCT 00397995. The current author did not receive any funding. The author of the primary study received a grant from the Anna Foundation, the Netherlands. None of the authors declare any conflicts of interest.

## Results

Between 2006 and 2010, 66 children were included in the RCT by Colaris et al. (2012) and 51 of these children participated in the current study: 26 out of 31 patients who were allocated to AEC, and 25 out of 35 patients who were allocated to BEC. The mean length of follow-up was 7.3 years (5.9–8.7). Baseline characteristics of the groups and primary outcome, the loss of forearm rotation, showed no statistically significant differences between the 2 treatment groups (Tables 1 and 2) Secondary outcomes were similar between the 2 treatment groups (Table 3). No statistically significant differences were found in sagittal or coronal angulation of the radius and ulna in either group (Table 4). The interrater reproducibility of the radiological assessment showed an intra-class correlation of 0.83 (CI 0.57–0.94).

**Table 1.** Baseline characteristics of the population. Values are count unless otherwise specified

Baseline	Below elbow cast	Above elbow cast
Number of children	25	26
Age at trauma (SD)	7.5 (1.4)	6.2 (1.4)
Male sex	12	10
Dominant arm	5	10
Type of fracture, radius		
Buckle	0	2
Greenstick	16	17
Complete fracture	9	7
Type of fracture, ulna		
Buckle	2	4
Greenstick	19	19
Complete fracture	4	3

**Table 2.** Representation of follow-up population

	Lost to FU	Included	Total
	(95% CI)	(95% CI)	(95% CI)
Outcome at 7 months follow-up	n = 15	n = 51	n = 66
Age at trauma, years	7.9 (6.1–9.8)	6.8 (5.8–7.8)	7.1 (6.2–7.9)
Male sex, n	8	29	37
Forearm rotation 7 months (°)	148 (144–153)	139 (131–148)	146 (142–150)
Loss of rotation (°)	4.9 (2.9–6.9)	4.3 (0.6–8.1)	4.8 (3.1–6.5)
ABILHAND-kids questionnaire (points)	41.4	41.7	41.6
Complications (%)	13	16	15
VAS cosmetics parents/child (0–10)	9.6	9.4	9.4
VAS cosmetics surgeon (0–10)	9.8	9.7	9.7

**Table 3.** Data (95% confidence intervals) on primary and secondary outcomes at long-term follow-up

	Below elbow cast	Above elbow cast
Factor	n = 25	n = 26
Age at follow-up, years	14.9 (13.3–16.5)	13.2 (11.8–14.7)
Follow-up length, years	7.5 (6.9–8.0)	7.1 (6.5–7.7)
Loss of forearm rotation	–0.72 (–4.5 to 3.1)	0.58 (–5.1 to 6.2)
Loss of wrist flexion–extension	0.80 (–3.2 to 4.8)	0.58 (–2.4 to 3.5)
ABILHAND-kids		
questionnaire (points)	41.4 (40.1–42.7)	41.9 (41.5–42.3)
DASH score (points)	4.4 (0–13)	2.1 (0–6.9)
Grip strength, kg	31 (17–45)	28 (14–42)
VAS cosmetics parents (0–10)	9.4 (9.0–9.8)	9.3 (8.9–9.7)
VAS cosmetics surgeon (0–10)	9.7 (9.5–9.9)	9.6 (9.4–9.9)

**Table 4.** Radiological angulation (°) (95% confidence intervals)

View	Below elbow cast		Above elbow cast	
	Cast removal	Final	Cast removal	Final
Anteroposterior				
Ulna	7 (2–12)	5 (2–8)	6 (2–10)	5 (2–8)
Radius	4 (0–8)	6 (3–9)	4 (0–8)	5 (1–9)
Lateral				
Ulna	6 (2–10)	4 (1–7)	6 (3–9)	4 (2–6)
Radius	10 (5–15)	5 (2–8)	10 (5–15)	4 (1–7)

Cast removal at 6 weeks after casting

## Discussion

We present the results of a multicenter randomized controlled study with 7-year follow-up, concerning children with a minimally displaced metaphyseal both-bone fracture of the distal forearm who had been treated with either AEC or BEC. Short-term follow-up of these patients at 7 months showed no statistically significant differences, except more cast comfort and less interference with daily activities in the group treated with BEC. The 7-year follow-up revealed similar outcome between the 2 groups concerning loss of forearm rotation, patient-reported outcome measures (PROMs): DASH and ABILHAND-kids questionnaire, grip strength (using a JAMAR dynamometer), VAS scores regarding cosmetic appearance (scars and angulation of the forearm), and radiological assessment of malunion.

### Previous research with short-term follow-up

A meta-analysis by Hendrickx et al. (2011) included 3 RCTs comparing AEC with BEC for the treatment of both-bone distal forearm fractures in 219 children. Secondary fracture displacement was seen in 15% in the BEC group and in 28% in the AEC group. An update of this meta-analysis, which included 2 more studies with 174 more children, found no treatment preference any longer. Concerning the plaster-related complication rate, data was pooled and showed no difference between the 2 treatment strategies (Van Den Bekerom et al. 2012).

### Previous research with long-term follow-up

Literature on long-term follow-up of nonoperative treatment of forearm fractures in children is scarce. A retrospective study of Zimmerman et al. (2004) included 220 children with 232 distal forearm fractures between 1980 and 1992. The mean age of included children was 9 years (1–16) and the mean time of follow-up 10 years (5–16). In 40 children both the radius and ulna were fractured. The purpose of this study was to investigate the frequency and extent of clinical and radiological late sequelae and identify predicting factors. The overall outcome was very good in 72%, and children < 10 years of age showed more favorable results, even with a malunion. Children > 10 years of age with an angulatory deformity > 20 degrees and/or more than 50% displacement at consolidation showed more pain and less function. Further factors having a negative influence on the outcome were repeated reduction and an additional fracture of the ulna.

We would like to address the ongoing debate on how much fracture angulation can be accepted at what age. The highest remodeling capacity is expected in young children with fractures close to the most active distal growth plate, and angulation in the sagittal plane. However, the literature on acceptable angulation in pediatric forearm fractures is scarce. Ploegmakers and Verheyen (2006) carried out a meta-analysis and together with the opinions of 18 international experts an effort was made to provide insight into the limits of acceptance of angular deformation in the nonoperative treatment of pediatric forearm fractures. More specifically for metaphyseal both-bone fractures of the distal forearm, the literature showed acceptable angulation of 11–18°, compared with 6–24° by the experts. Our primary inclusion criteria (fracture angulation < 15° in children < 10 years and < 10° in children ≥ 10 years) were in the range of these results. Our good clinical and radiological long-term follow-up results combined with previous literature (Ploegmakers and Verheyen 2006, Prommersberger and Lanz 2000) shows that metaphyseal both-bone fractures of the distal forearm especially in children < 12 years of age remodel satisfactorily (Prommersberger and Lanz 2000).

### Study limitations

Our main limitation is the long-term follow-up percentage of only 77% of the primarily included children, but previous literature on acceptable loss to follow-up suggests that up to 40% loss to follow-up results in minimal attrition of the results (Kristman et al. 2004, Fewtrell et al. 2008). To address the potential effects of loss to follow-up we did a patient group analysis, which showed that the follow-up group was representative of the whole original study group.

Limitations of the original study still apply. The reduction criteria were adjusted to only 2 age groups without gender distinction, and the reduction criteria were used for both metaphyseal and diaphyseal forearm fractures. Therefore, the reduction criteria for metaphyseal fractures in the youngest children, especially boys, could probably have been too strict.

## Conclusions

At long-term follow-up we found similar loss of forearm rotation after treatment of minimally displaced metaphyseal both-bone fractures of the distal forearm in children treated with AEC or BEC. Furthermore patient-reported outcome measures and radiological assessment were similar. Based on short- and long-term results, we suggest that children with minimally displaced metaphyseal both-bone forearm fractures should be treated with a below-elbow cast.
